# Income Inequality, Neighbourhood Social Capital and Subjective Well-Being in China: Exploration of a Moderating Effect

**DOI:** 10.3390/ijerph18136799

**Published:** 2021-06-24

**Authors:** Jiawen Huang, Yitong Fang

**Affiliations:** School of Public Administration, South China University of Technology, Guangzhou 510641, China; 202020133173@mail.scut.edu.cn

**Keywords:** subjective well-being, income inequality, neighbourhood social capital, moderating effect, multilevel analysis

## Abstract

With the continuous global rise in inequality and the growing importance of subjective welfare, the relationship between income inequality and subjective well-being has received increasing attention. This paper focuses on neighbourhood social capital, measured at the individual and community levels, to explore its moderating effect on the association between income inequality and subjective well-being in the context of China, an issue few studies have examined. Using data from the China Labour-force Dynamics Survey and multilevel models, the results show that income inequality measured using three different indicators had a stable and negative association with subjective well-being in China, after controlling for various individual characteristics and aggregate-level factors. Although neighbourhood social capital at the individual level has been proven to promote subjective well-being, a dark side of social capital is also found at the community level. More notably, neighbourhood social capital at the individual level can attenuate the negative impact of income inequality on subjective well-being, especially for vulnerable groups, such as those with low income or low education. How to reasonably guide the community to develop social capital is an important policy implication to attenuate the negative psychological experience of income inequality.

## 1. Introduction

Subjective well-being refers to positive feelings and attitudes, which mainly consist of individual emotions and life evaluations [1]. Not only is subjective evaluation of the quality of life at the individual level important, but it is also a key indicator to evaluate whether social policies are able to meet people’s needs at the national level [2]. Since the 1950s, the study of subjective well-being has increasingly become a theoretical focal point within the global academic context, arousing widespread discussion in psychology, economics, sociology, and many other disciplines. One well-known finding is the ‘Easterlin Paradox’, which indicated that there was no significant difference in the average level of subjective well-being between rich and poor countries. In terms of the diachronic effect, it was claimed that economic development has not brought about an improvement in national subjective well-being [3]. One of the most important explanations focuses on the economic field and seeks to develop relevant theories concerning habituation effects, relative income and income inequality [4].

The investigation of income inequality in relation to subjective well-being began later. With the continuous global rise in income inequality and the increasing concerns in numerous countries [5], and with measures of subjective welfare growing in popularity within the social sciences [6], the relationship between income inequality and subjective well-being has recently attracted increasing attention. As a case in point, in China, income inequality has increased sharply in the last three decades, with the Gini coefficient exceeding 0.5 in around 2010 [7], and remaining between 0.47 and 0.48 in 2010–2018 (Data source: 6. National Bureau of Statistics, China yearbook of household survey 2019, Beijing China: China Statistics Press, 2019). This has led to the rapid increase in studies on the relationship between income inequality and subjective well-being. However, the effect of income inequality on subjective well-being remains controversial, with no consensus on the strength and direction of the relationship between them in an empirical analysis [8,9,10,11]. One recent study has argued that the lack of attention to the various social processes intertwined with income inequality and subjective well-being is the main cause of the mixed findings [12]. It is insufficient to simply find a correlation between income inequality and subjective well-being; instead, a plausible theoretical explanation is needed to address this issue in terms of causation. Covariation with other economic and social conditions that alter or modify relevant links should be included in the analysis.

In studies of the influencing mechanisms, neighbourhood social capital is a key factor that cannot be ignored. With a growing number of studies on the spatial variation in subjective well-being, the neighbourhood, as an important space of daily life that significantly shapes individual living environments and life chances, has gained importance in analysing subjective well-being [13]. With changes in the social division of labour and the development of communication technology, no consensus has emerged on whether the degree of social interaction among neighbours is weakening or being maintained [14,15], with competing claims made concerning communities, ranging across the ‘liberated’, ‘lost’, and ‘saved’ perspectives [16]. However, a recent study has reiterated the transformative potential of group-based social connection, which could not be reduced to those group members functioning as individuals. Moreover, it was important to explain health and well-being [17], which was also crucial in the neighbourhood. Haslam et al. [18] discovered that joining community groups could enhance people’s well-being by strengthening their group identities and bringing a broader sense of belonging to multiple groups. Other empirical studies have also reported that the neighbourhood, as a form of social capital, has a positive relationship with subjective well-being [19,20], specifically in China. On the one hand, some studies have pointed out that social capital established through geographic proximity and consanguinity still plays an important role in information sharing and resource allocation in China [21]. On the other hand, the community, as the most basic unit of state-led social governance in China, provides stable and strong institutional constraints for the maintenance or reconstruction of neighbourhood relationships. Given this context, it is likely to be worthwhile to introduce neighbourhood social capital into the study of the mechanisms connecting income inequality and subjective well-being. So this paper examined whether income inequality and neighbourhood social capital were associated with subjective well-being, and whether neighbourhood social capital moderated the relation between income inequality and subjective well-being in the Chinese context.

## 2. Literature Review and Research Hypotheses

### 2.1. Income Inequality and Subjective Well-Being

The pioneering study of Morawetz et al. [22], which indicated that living in a more egalitarian community facilitated happiness, generated extensive studies on the relationship between income inequality and subjective well-being (happiness and life satisfaction), but no consensus has been reached. Studies have found a negative effect [23], a positive effect [24], or no significant effect [25].

Accurately estimating the effect of income inequality on subjective well-being remains challenging. Although some studies lack clear theoretical explanations, the implied theoretical standpoint held by the various authors can be determined. Findings supporting a positive effect of income inequality on subjective well-being have been explained using the concept of a ‘tunnel effect’ [26]. The original use of the concept referred to traffic jams in two-lane tunnels. When a car in one lane starts to go more slowly, the drivers in the other lane have an optimistic attitude concerning their own lane, even if their lane is still impeded. Similarly, income inequality implies differences in income, which can signal opportunities for upward mobility or greater fairness, and allow people to have expectations of benefiting from the changing income distribution instead of being confined to their current social status [27]. In line with this perspective, Alesina et al. [28] found that Europeans, when compared with Americans, felt that they were living in a less mobile society and were more sensitive to the negative effect of income inequality on happiness.

Studies showing the negative effects of income inequality on subjective well-being have formed the following two explanatory mechanisms: an external environment mechanism and a social psychological mechanism. Concerning the first mechanism, it has been claimed that income inequality, possibly producing poor living conditions involving high crime rates and the loss of social capital, in turn reduces subjective well-being. This perspective essentially reflects the individual unhappiness caused by the mismatch between the institutional setting and individual needs. A growing number of studies have provided evidence that income inequality lowers social trust, leading to ineffective social support systems, which have a negative effect on life satisfaction in different regions [29]. Concerning the second mechanism, studies have focused on the underlying social psychological mechanisms for the link between income inequality and subjective well-being. Excessive income gaps are claimed to violate people’s preference for fairness, increase a sense of relative deprivation compared with others [30], or lead to status anxiety, especially for vulnerable groups [31], which then affects the individuals’ subjective well-being.

In studies in the Chinese context, the effect of income inequality on subjective well-being has been found to be mixed and inconsistent [8,9,10,11]. In addition to the different data used in these studies, the critical factor of rapid social change in a transitional period needs to be considered. Following reform and the opening up of the Chinese economy, the development of the market economy broke up the original pattern of equalized income distribution and provided an important opportunity for people to change their social status. However, some studies have shown that class background continues to play a key role in people’s access to education and advances in their professional status [32]. This factor has become more important, as class immobility in China has been shown to have increased from 1996 to 2012 [33] and to be profoundly influenced by social structural factors, such as regional political and socioeconomic conditions [34]. Given this situation, income inequality may no longer be a positive signal of hope and an expectation of upward mobility, especially in the context of reinforced class solidification. Furthermore, social problems, including environmental pollution, health-compromising behaviour and the burden of steeply increased housing costs, have been shown to be negative consequences of income inequality in China [35,36], and important factors altering the quality of the living environment and reducing subjective well-being. Accordingly, this paper evaluated the following hypothesis:

**Hypothesis** **1.** *Income inequality decreases subjective well-being.*

### 2.2. Neighbourhood Social Capital and Subjective Well-Being

Neighbourhood social capital, which has been defined as a special kind of social support from people who live in close proximity or public resources facilitating action and cooperation for common interests in the neighbourhood [37], is widely believed to play an important role in subjective well-being, including happiness, life satisfaction, and emotional response [38,39]. Generally speaking, it is divided into individual and collective components [40]. The individual component involves individual resources embedded in relationships with people living nearby. The collective component emphasizes the features of a community, such as the norms of reciprocity, civic involvement and interpersonal trust. Recently, in order to improve the accuracy of measurement and estimation, more and more empirical studies have favoured measuring neighbourhood social capital at the individual level and the community level to analyse the impact of individual and collective components on subjective well-being [20,41].

In terms of the individual component, the positive effect of neighbourhood social capital in the provision of emotional, instrumental and informational forms of social support in relation to subjective well-being has been examined in numerous studies [42,43]. Based on the theory of social support, there are two main mechanisms to explain the impact of neighbourhood social capital on subjective well-being. The stress-buffering model points out that the effect of social support can only be highlighted when a person is in a state of high stress. Social support is often defined as “providing psychological and material resources to improve an individual’s ability to cope with stress” [44]. One major study, which looked at 49 countries using different and wide-ranging sources of survey data, found that people living with friendly neighbours were less likely to have emotional problems [19]. This finding indicated that neighbourhood social capital worked as a stress buffer that could enhance subjective well-being by reducing disturbing negative effects. The main effect model holds that social support can make individuals remain healthy and feel good in all living environments, not just under high pressure [44]. Bartolini et al. [45] found a similar direct effect in the US over a 30-year period and concluded that the decline in social connections, including contact with neighbours, was the main reason for the decrease in happiness among Americans over time.

In terms of the collective component, attention has shifted to seeking understanding on how and why neighbourhood social capital with collective characteristics influences individuals’ subjective well-being. On the one hand, in line with the theory of social support, being in a well-connected neighbourhood reflects the convenience of access to resources on a larger scale, which has been found to have a positive effect on improving the subjective well-being of residents [20]. On the other hand, the collective component of neighbourhood social capital can refer to the social cohesion within the neighbourhood [46], which has been deemed to be a crucial aspect indicating the liveability of the social environment. A close-knit neighbourhood can generate a high level of informal social control in the community, which is indicated by the willingness of the residents to intervene under various conditions of crisis, and has a positive relationship with mental health and life satisfaction [47]. Therefore, this study evaluated the following hypotheses:

**Hypothesis** **2.** *Neighbourhood social capital at the individual level increases subjective well-being.*

**Hypothesis** **3.** *Neighbourhood social capital at the community level increases subjective well-being.*

### 2.3. Income Inequality, Neighbourhood Social Capital and Subjective Well-Being

Social capital, mainly referring to social trust, has been shown to be an important mechanism that influences the relationship between income inequality and subjective well-being. Oishi et al. [29], using US General Social Survey data from 1972 to 2008, found that income disparity led to divisions among members of society and created distrust, which reduced people’s happiness. Similar conclusions were drawn by Delhey and Dragolov [48] in an empirical study in Europe. These results indicated that inequality made it difficult to form a sense of togetherness, which undermined trust and was not conducive to improving happiness. However, these studies did not include the geographical scope in the definition of social capital, and the moderating effect of social capital on income inequality and subjective well-being was not verified, which may lead to misinterpretation of the real relationship between them. This study considered that the negative influence of income inequality on subjective well-being can be mitigated through neighbourhood social capital for the following reasons.

At the individual level, according to the social support theory, people may obtain emotional support through harmonious neighbourhood interaction, which can relieve the negative feelings caused by inequality, such as aversion, dissatisfaction or pressure. Neighbourhood social capital works as an important buffer against negative emotions, and inhibits psychological anxiety and depression [19]. In addition, neighbourhood social capital might offer particular resources, such as employment information or solutions to difficulties for material gain, which could attenuate the effect of income inequality on subjective well-being by helping improve individual economic conditions.

A personal network with neighbours provides access to community-level social capital [49], which means that people can share emotional and material resources more widely, and can create a harmonious and stable environment for people to express their negative feelings and worries [50]. Furthermore, as Moore et al. [51] found, greater neighbourhood interaction signified the availability of more resources and a greater need for individuals to closely adhere to community norms. In other words, neighbourhood interaction was a potentially powerful way to create solidarity and emotional cohesion in a neighbourhood, fostering individual integration into the community. The norms fostered through close community connections guide individuals to seek collective support when facing difficulties, and operate as a form of social control to mitigate individual irrational behaviour caused by negative emotions. Given these considerations, this paper evaluated the following hypotheses:

**Hypothesis** **4.** *Neighbourhood social capital at the individual level attenuates the effect of income inequality on subjective well-being.*

**Hypothesis** **5.** *Neighbourhood social capital at the community level attenuates the effect of income inequality on subjective well-being.*

## 3. Methodology

### 3.1. Data and Sample

This study used data obtained from the China Labour-force Dynamics Survey (CLDS) in 2014, which was organized by the Centre for Social Science Survey at Sun Yat-sen University in Guangzhou. The CLDS was a major interdisciplinary nationwide survey with a large sample size that focused on the current situation and changes in the labour force in China and covered many research topics, such as work, migration, health, and social participation. Compared with other nationwide surveys, this survey offers numerous advantages, one being its data collection from the three levels of the individual, family and community, which facilitates extensive research into the interactive effects of factors at these levels on the labour force and the use of multilevel analysis. To ensure sample representativeness, the CLDS used multistage cluster stratified probability proportionate to size sampling. Face-to-face interviews with 23,594 respondents in 29 mainland provinces and municipalities (except the provinces of Tibet and Hainan) were conducted by interviewers who were well trained and familiar with computer-assisted interviewing techniques.

For this study, a subsample was selected from the total sample, which included 18,869 respondents. The selection criteria were as follows: (1) the age of the respondents ranged from 15 to 64 years old, and (2) respondents should have work experience, referring to the engagement in remunerative activities such as farming, part-time jobs and helping with family businesses. Students, volunteers and those engaged in housework were excluded. The final sample comprised 15,501 respondents in 29 mainland provinces (including 391 communities and 123 cities), following the exclusion of respondents with missing values concerning demographic variables and key factors affecting subjective well-being.

As shown in Table 1, the male-to-female ratio of the sample was nearly 1:1. More than half of respondents were between 40 and 60 years old. Further, 87.82% of respondents were married, and 74.19% of respondents had primary or high school education. Most respondents’ registered permanent residence was located in the county where the survey was conducted.

### 3.2. Measures

*Dependent variable.* Although subjective well-being is a multidimensional concept, the measurement of self-reported happiness with a single item rated on a multipoint scale has been found to be a reliable and valid method [52] when compared with multi-item instruments assessing participants’ subjective well-being. Studies using this method have shown a strong association between subjective well-being and positive effects, such as self-esteem, optimism, and good mental health [53], and the method has been widely used in studies on subjective well-being [8,46]. Therefore, a single question, ‘How do you feel about your life?’, measured on a 5-point Likert scale ranging from 1 (very unhappy) to 5 (very happy), was used to evaluate subjective well-being.

*Independent variable.* The diversity of indicators for measuring income inequality in previous studies is striking. To fully reflect regional income inequality and enhance the reliability of the results, this study used multiple indicators, including the Gini coefficient, the P90/P50 and the Theil index, to measure income inequality. The three indicators at the aggregate level were calculated by taking specific regions as basic units, which is a recognized approach in the analysis of the relationship between income inequality and subjective well-being [8,9]. In line with previous studies, the results using the three indicators were calculated by aggregating micro-individual income data within 123 surveyed cities, referring to the administrative division of the People’s Republic of China (PRC) ranking between a province and a county and including the central urban areas and the surrounding rural areas.

Neighbourhood social capital was measured at both the individual and the community levels. Because social capital is a remarkably rich concept involving multiple definitions and types of operationalization [54], systematic measurement of neighbourhood social capital has been lacking. According to precedents, we measured neighbourhood social capital from a social cohesion-based perspective, which defines social capital as the level of quality and trust of interpersonal relationships among community members [55]. Questions including ‘Are you familiar with your neighbours?’, ‘Do you trust neighbours?’ and ‘Do you help your neighbours?’ were used to measure neighbourhood social capital at the individual level. The respondents were asked to respond to these items on a scale ranging from 1 to 5. A higher score calculated using the three items (ranging between 3 and 15) represented a higher level of neighbourhood social capital. Cronbach’s alpha (α = 0.82) showed that the three items were internally consistent. Neighbourhood social capital at the community level was measured by aggregating individual responses (including familiarity with neighbours, trusting neighbours and helping neighbours on a scale ranging from 1 to 5) and defined as the sum of the proportions of those who answered 4 and 5 of the above three questions in each community. Although there has been some controversy about the validity of these measures, we still adopted them based on scholars’ support and use of them [55,56].

*Control variables.* Given that subjective well-being is a complex mental state influenced by personal factors and social conditions, several control variables at different levels needed to be taken into account to reduce the risk of statistical bias. Through a review of previous studies, it was found that individual characteristic variables, such as gender, age, age2, marriage status, registered permanent residence, education, political status, work status, sense of fairness, log of income and subjective social status, and aggregate-level variables, such as the security and population size of the community as well as economic growth and regional income levels, are significant predictors of subjective well-being [57,58]. This study incorporated the above variables into its model analysis. Descriptive statistics of the specific variables are shown in Table 2.

### 3.3. Statistical Strategy

Since the dependent variables of this study concerned variables at the individual level and the core independent variables included variables at the aggregate level, multilevel analysis was applied. Multilevel analysis is used to explain the spatial dependence of individuals in certain regions and estimate effects originating from different levels. A three-level linear random intercept model was considered appropriate, where individuals (15,501) were nested within communities (391) that in turn were nested within cities (123). Statistical software used in this study was Stata 12.0. The linear multilevel equation of this study is shown in the following formula:

(1) $$Y=\left(\gamma_{0}+\gamma_{1}X_{\mathrm{ijk}}+\gamma_{2}X_{.\mathrm{jk}}+\gamma_{3}X_{\ldots k}\right)+\vartheta (\mathrm{Aggregate}\;\mathrm{Income}\;\mathrm{Inequality}\;\times \mathrm{Neighbourhood}\;\mathrm{Social}\;\mathrm{Capital})+(e_{\mathrm{ijk}}+e_{\mathrm{jk}}+e_{k})$$

where Y denotes the self-reported happiness of the respondent X_.jk_ and X_..k_ are the means of predictors in the level-2 and level-3 units, respectively. X_ijk_ is included to account for observed sources of variation in the responses at the individual level. ϑ  is the interaction effect between aggregate income inequality and neighbourhood social capital. γ_0_ is a random intercept, and γ_1_, γ_2_, γ_3_ are the coefficients at different levels. e_ijk_, e_jk_, e_k_ indicate residuals that are assumed to be normally distributed and uncorrelated with the predictors included in the model.

## 4. Results

### 4.1. The Effect of Income Inequality and Neighbourhood Social Capital on Subjective Well-Being

Table 3 shows the effect of income inequality and neighbourhood social capital on subjective well-being, after controlling for variables at the aggregate level and personal characteristics. The intraclass correlation coefficient (ICC) of the null model demonstrated that 5.4% and 6.7% of the respondents’ variance in subjective well-being originated at the city level and community level, respectively. When income inequality, neighbourhood social capital, and other potential confounding variables were all included, as shown in Model 3 of Table 3, the ICC coefficient (ICC = 0.020) at the city level decreased by 63%, while the ICC coefficient (ICC = 0.053) at the community level decreased by 21% compared with that of the null model. The proportional reduction in residual variance at the individual level was between 15.9% and 17.1%. These results showed that estimation using a multilevel model had significant advantages for precise calculation.

The relationship between income inequality and subjective well-being was consistent with the expectations. The results of Model 1 showed that the Gini coefficient was highly correlated with subjective well-being at the 0.1% significance level when controlling for key variables at different levels. The same situation was found in Model 3, with a coefficient of −0.674 (*p* < 0.01). These results implied that increasing income inequality at the city level was related to lower levels of individual subjective well-being. The data results supported Hypothesis 1.

However, the effects of neighbourhood social capital, at different levels, on subjective well-being were different. On the one hand, the results of Models 2 and 3 showed that neighbourhood social capital at the individual level was highly correlated with subjective well-being at the 0.001 significance level, when other variables were controlled. On the other hand, the effect of neighbourhood social capital at the community level on subjective well-being was negative (b = −0.097, *p* < 0.001; b = −0.085, *p* < 0.001). These results only supported Hypothesis 2, not Hypothesis 3.

In terms of control variables, the results of Model 3 also showed an association between subjective well-being and the relevant variables at different levels. Men were more likely to feel unhappy than women (b = −0.057, *p* < 0.001). There was a U-shaped relationship between age and subjective well-being at the 0.1% significance level (b = −0.033, b^2^ = 0.033, *p* < 0.001). The subjective well-being of married people was found to be higher than that of unmarried people (b = 0.251, *p* < 0.001). Education (b = 0.015, *p* < 0.001) and political status (b = 0.102, *p* < 0.001) were closely related to subjective well-being. It also appeared that improving the sense of fairness would be likely to promote personal perceived happiness (b of neutral = 0.153, b of fair = 0.456, *p* < 0.001). The link between subjective well-being and local household registration, which indicated the differences in resource and welfare distribution between locals and outsiders, was found to be strong (b = 0.077, *p* < 0.01).

In terms of economic factors, subjective social status had a significant association with subjective well-being (b = 0.101, *p* < 0.001). However, there was no significant association between the logarithm of income and subjective well-being. Local GDP per capita had a weak positive effect on subjective well-being, as shown in Model 2 (b = 0.002, *p* < 0.05), while the local income level was correlated with subjective well-being. At the community level, community security had a significant positive effect on individuals’ subjective well-being (b = 0.069, *p* < 0.01). The population size of the community was not found to have a significant relationship with subjective well-being.

### 4.2. Robustness Checks

After controlling for the key variables at the different levels, both the Theil index (b= −0.238, *p* < 0.01) and the P90/P50 (b = −0.023, *p* < 0.05) were highly associated with subjective well-being, which indicated that income inequality had a significant negative relationship with subjective well-being, as reported in Table 4. Additionally, the effect of income inequality on subjective well-being was significant in two further models. On the one hand, life satisfaction as an alternative dependent variable in relation to income inequality was incorporated into a hierarchical linear model. The results showed that the Gini coefficient had a significant negative association with life satisfaction (b = −0.549, *p* < 0.01). On the other hand, subjective well-being was combined into a binary dependent variable (respondents with responses of 4 or 5 were categorized as ‘happy’; others were categorized as ‘unhappy’), and a multilevel logit model was used to estimate the relationship with income inequality. Although the significance of the results decreased, there was still a negative relationship between income inequality and subjective well-being (odds ratio = 0.229, 95% CI = 0.062~0.841).

This study adopted the same strategy of robustness checks to estimate the relationship between neighbourhood social capital and subjective well-being. As shown in Table 4, when controlling for individual characteristics and several key variables at the aggregate level, neighbourhood social capital variables at the individual level had a positive subjective relation, while a negative effect of neighbourhood social capital variables at the community level was found in all models. Therefore, the overall results indicated that the effects of income inequality and neighbourhood social capital on subjective well-being were stable and significant.

### 4.3. The Moderating Effect

This study further explored whether the interaction effect of neighbourhood social capital and income inequality at the individual and community levels affected individual subjective well-being. At the individual level, as shown in Table 5, after controlling for individual characteristics and some key variables at the community level and at the city level, the interaction coefficient between the Gini coefficient and neighbourhood social capital was 0.083, which was significant at the level of 0.05. Furthermore, the interaction between the other two indicators measuring income inequality (the Theil index and P90/P50) and neighbourhood social capital was found to have a significant positive effect on subjective well-being (b = 0.037, *p* < 0.05; b = 0.004, *p* < 0.05). These results showed that neighbourhood social capital at the individual level could effectively attenuate the negative effect of income inequality on subjective well-being; thus, Hypothesis 4 was supported.

In contrast, this study found differing results concerning neighbourhood social capital at the community level. The interaction between the Gini coefficient, the Theil index, P90/P50 and neighbourhood social capital was not significant when the control variables were included at the community level, as reported in Table 5. In other words, neighbourhood social capital at the community level did not attenuate the negative effect of income inequality on subjective well-being; thus, Hypothesis 5 was not supported.

### 4.4. Subsample Analysis

Previous studies have shown that the importance of neighbourhood social capital varies from person to person, especially for the economically disadvantaged, the elderly, and the unemployed, who are more dependent on the support of neighbours and neighbourhood institutions, due to the lack of time and personal resources [46]. Therefore, it was necessary to further investigate the moderating effect in different subgroups. Because the subsamples of the people over 60 years old (n = 1879) and the unemployed (n = 1098) were small, they could not meet the requirements of using multilevel models. We just considered the differences in economic conditions, and investigated the moderating effect on different education levels and income levels. The subsample results are shown in Table 6.

The results of Model 11 and Model 12 in Table 6 show that when the key control variables were included, the impact of income inequality on subjective well-being was different between people with low education and those with high education. The negative effects of income inequality existed only for people with low education (b = −1.619, *p* < 0.05). Neighbourhood social capital at the individual level could slightly attenuate the negative effect of income inequality on subjective well-being, especially for people with a low level of education (b = 0.074, *p* < 0.10). However, the interaction effect of neighbourhood social capital at the community level had no significant relationship with subjective well-being.

For different income groups, there was a significant negative relationship between income inequality and the subjective well-being of poor individuals (b = −1.768, *p* < 0.05), which was not observed for rich individuals. Neighbourhood social capital at the individual level could slightly reduce the negative impact of income inequality on subjective well-being for poor individuals (b = 0.095, *p* < 0.10). In contrast, the interaction effect of neighbourhood social capital at the community level was very weak, whether for poor or rich individuals.

## 5. Discussion

This study focused on neighbourhood social capital at different levels and expanded the understanding of the mechanism connecting income inequality and subjective well-being, based on the CLDS data from a representative nationwide survey. 

The findings from this study demonstrate that income inequality shows a significant and stable negative correlation with individuals’ subjective well-being. This is not consistent with other studies conducted in China, which indicate that income inequality has a significant positive relationship [8] or an inverted U-shaped relationship [9] with subjective well-being. The differences are likely due to the rapid social change occurring in China. Since the reform and opening up, especially after the establishment of the market economic system, the principle of meritocracy has become the main standard for selecting talent in the labour market, which has weakened the intergenerational transmission of social status and enhanced social mobility in China [59]. At that time, income inequality was regarded as a positive signal, offering the prospect of upward mobility and the possibility of changing one’s social status.

In recent years, China has gradually formed a relatively stable pattern of vested interest groups, and strengthening intergenerational inheritance and impeding intergenerational mobility have become important means for safeguarding vested interests, which has led to a stronger impact on the reproduction mechanism of the whole social class. Some empirical results show that the effect of class background is becoming more significant, reflecting a tread towards growing class immobility [33]. Therefore, income inequality is more readily deemed to be a negative signal, leading to lower individual subjective well-being. Furthermore, increasingly serious social problems, caused in part by income inequality, such as the burden of high housing costs, tend to arouse dissatisfaction, which further diminishes the likelihood of reporting experiences of happiness. The above explanation implies that the relationship between income inequality and subjective well-being should be discussed in the context of different countries, rather than being limited to a meaningless statistical game [60].

This study also shows that neighbourhood social capital at the individual level can significantly improve subjective well-being, while a negative effect of neighbourhood social capital is found at the community level. It implies that the dark side of the effect of neighbourhood social capital on subjective well-being needs to be given more attention, which is different from previous studies’ emphasis of a positive impact. Those studies pointed out that higher neighbourhood social capital leads to a higher degree of social organization, which not only provides instrumental support for residents, but also influences psychological processes by providing emotional support and enhancing self-esteem to support subjective well-being [19,20]. However, similar to the potential negative impact of social capital put forward by Portes [40], a group based on close ties may place excessive demands on its members, exerting considerable pressure on the members and preventing them from acting according to their own wishes because of strong norms and constraints. In China, due to the influence of traditional culture, which emphasizes the importance of social networks, neighbourhood social capital still plays an important role in residents’ daily lives. However, living in a community with high neighbourhood social capital also indicates the fulfilment of more responsibilities and obligations, and the constraints of community norms. This kind of close and frequent neighbourhood interaction often becomes an important source of psychological pressure [61,62], especially for residents living in rural communities, whose daily life is limited within the boundaries of the village. It is also difficult to ignore the pressure posed by community members who come from outside the private sphere, but have the ability to penetrate into private life, which can reduce individual subjective well-being.

Another contribution of this study compared with previous studies concerns the moderating effect of neighbourhood social capital [29,48]. It is found that neighbourhood social capital is able to attenuate the negative effect of income inequality on subjective well-being, but this moderating effect is present only in relation to the variables at the individual level. Changes in the division of labour, as well as the development of electronic communication, have allowed individual social interaction to move beyond the scope of one’s living space. This development has changed the shape of communities and individual social networks, which have become loose, diversified, weak, and diffused in space. The wider geographical spread of social interaction also means that neighbourhood interaction has become a more voluntary activity, in which residents have greater freedom of choice in terms of whom they interact with and where [63]. It has been claimed that only voluntary participation in neighbourhood activities, rather than compulsory participation, can become a means to improve subjective well-being [64]. Neighbourhood social capital at the individual level involves the exercise of personal choice, which enables a positive and active process in constructing social networks. Therefore, its moderating effect on subjective well-being is likely to be stronger than the influence of a more passive embedded and pre-existing community environment.

Through the analysis of subsamples, this study further finds that the moderating effect of neighbourhood social capital is different among groups. Neighbourhood social capital at the individual level can attenuate the negative impact of income inequality on subjective well-being, especially for vulnerable groups, such as those with low income or low education. The relative importance of neighbourhood social capital for a person is decided by his or her networks outside the neighbourhood boundaries [46]. People who are economically disadvantaged, such as those with a low income or low education level, are often subject to great geographical restrictions in terms of social interaction [65]. This leads to their strong dependence on the community as the main source of information, resources, services, and opportunities. Therefore, certain types of networks, reciprocity and trust formed as part of neighbourhood social capital are likely to have a stronger influence on the relationship between income inequality and subjective well-being for vulnerable groups than for other groups.

The results of this study show that neighbourhood social capital, as a norm embedded in the social structure and shared resources generated by social networks, can provide a potential intervention to attenuate the negative impact of regional income inequality on individuals’ subjective well-being. On the one hand, we can see that living in a community with more social capital, without appropriate interaction with a specific neighbourhood or moderate integration into the community network, is not enough to obtain positive social support. Therefore, how to mobilize the initiative of community members from the individual level and make them actively participate in neighbourhood communication and community activities is an important premise to cultivate neighbourhood social capital. On the other hand, compared with other groups, vulnerable groups in the community, such as people with a low income and education, are more vulnerable to the negative impact of income inequality, and are more dependent on the resources provided by the community. To cultivate neighbourhood social capital, more attention should be paid to the needs of these groups, and essential material and emotional support should be provided for them.

## 6. Limitations

This study has some limitations that need to be improved upon. Although measuring neighbourhood social capital at the community level, by aggregating individual responses, is widely accepted in most studies, this method has a few of the following shortcomings: it is affected by the individual characteristics of the respondents, and the reliability of the measure is easily influenced by the number of community respondents [66]. Ecological measurement methods will be used for further analysis, which account for the nesting of social capital items within individuals, integrate the individual and community levels to form a three-level model, and use the residuals at the community level to measure neighbourhood social capital. In addition, this study used cross-sectional data, which might lead to estimation errors due to unobserved individual differences or heterogeneity. Future studies need to use panel data to overcome this problem.

## 7. Conclusions

Despite these limitations, this study contributes to a body of research suggesting that the impact of neighbourhood social capital at different levels on subjective well-being has two sides. More importantly, neighbourhood social capital at the individual level plays an important role in alleviating the negative effect of income inequality on subjective well-being, especially for vulnerable groups. This finding will supports the understanding of the linkage mechanism between income inequality and subjective well-being.

## Figures and Tables

**Table 1:** Demographic characteristics of the samples.

Variables	Frequency	Percentage (%)
Gender		
male	8434	54.41
female	7067	45.59
Age		
15–20	260	1.68
21–30	2322	14.98
31–40	2975	19.19
41–50	4740	30.58
51–60	3325	21.45
60–65	1879	12.12
Marital status		
unmarried	1361	8.78
married	13,613	87.82
divorced	205	1.32
widowed	322	2.08
Education		
below primary school	1811	11.68
primary school	3871	24.97
high school	7629	49.22
college and higher	2190	14.13
Registered permanent residence		
in the county where the survey was conducted	14,173	91.43
outside the county	1328	8.57

**Table 2:** Descriptive Statistics.

Variable	Definition	Mean	Standard Deviation
Subjective well-being	Perceived happiness of the respondent from 1 to 5	3.705	0.892
*Income inequality*			
Theil index	Theil index of income within the city	0.518	0.210
Gini coefficient	Gini coefficient of income within the city	0.509	0.089
P90/P50	Ratio of income of the 90th percentile to that of the 50th percentile within the city	3.452	1.888
*Neighbourhood social capital*			
At the individual level ^a^	Scores calculated based on three items	10.913	2.436
At the community level ^a^	The sum of the proportions of those who answered 4 and 5 in each community	1.757	0.679
*Individual characteristics*			
Gender	1 = male; 0 = female	0.544	0.498
Age	Age of the respondent in years	44.810	12.859
Age2	Age square of the respondent in years	2173.338	1177.238
Marriage status	1 = married; 0= otherwise	0.878	0.327
Registered permanent residence	1= in the county where the survey was conducted; 0= otherwise	0.914	0.279
Education	Educational years of the respondent	8.619	4.405
Political status	1 = member of Chinese Communist Party; 0 = otherwise	0.091	0.287
Work status	1 = has a job; 0 = does not have a job	0.929	0.256
Sense of fairness	1 = unfair; 2 = neutral; 3 = fair	2.247	0.786
Log of income (ten thousand yuan)	Logarithm of individual income in 2013	9.077	2.704
Subjective social status	Self-evaluation of social status from 1 to 10	4.524	1.677
*Control variables at the city level*			
Local GDP per capita (thousand yuan/person)	The city’s GDP divided by the total number of people in the city	50.653	29.018
Local income level ^b^	Ratio of the annual per capita disposable income of rural or urban households in a city to that in the PRC	1.141	0.402
*Control variables at the community level*			
Security of the community	1 = safe; 0 = otherwise	0.889	0.313
Population size of the community	The total number of people actually living in the community	1777.884	3611.296

Note. ^a^ The specific measure is described in the Measures section. ^b^ The National Bureau of Statistics of the PRC calculates the annual per capita disposable income of urban areas and rural areas in a city. Therefore, different calculations of this index were used for individuals living in urban areas and in rural areas of the same city in this study.

**Table 3:** The effect of income inequality and neighbourhood social capital on subjective well-being.

	(1) Gini Coefficient	(2) Neighbourhood Social Capital	(3) Gini Coefficient + Neighbourhood Social Capital
			
Gini coefficient	−0.691 ***		−0.674 **
	(0.207)		(0.209)
Neighbourhood social capital at the community level		−0.097 ***	−0.085 ***
		(0.024)	(0.024)
Neighbourhood social capital at the individual level		0.048 ***	0.047 ***
		(0.003)	(0.003)
Gender (male = 1)	−0.050 ***	−0.058 ***	−0.057 ***
	(0.013)	(0.014)	(0.013)
Age	−0.029 ***	−0.033 ***	−0.033 ***
	(0.003)	(0.003)	(0.003)
Age2/100	0.030 ***	0.033 ***	0.033 ***
	(0.003)	(0.003)	(0.003)
Marriage (married = 1)	0.250 ***	0.250 ***	0.251 ***
	(0.022)	(0.022)	(0.022)
Registered permanent residents (in the county where the survey is conducted = 1)	0.117 ***	0.074 **	0.077 **
	(0.028)	(0.027)	(0.027)
Education	0.015 ***	0.015 ***	0.015 ***
	(0.002)	(0.002)	(0.002)
Political status (member of the Communist party = 1)	0.116 ***	0.102 ***	0.102 ***
	(0.024)	(0.023)	(0.023)
Work status (has a job = 1)	0.026	0.016	0.017
	(0.025)	(0.025)	(0.025)
Sense of fairness (reference group: unfair)			
neutral	0.154 ***	0.153 ***	0.153 ***
	(0.018)	(0.018)	(0.018)
fair	0.464 ***	0.456 ***	0.456 ***
	(0.017)	(0.017)	(0.017)
Log of income	0.004	0.003	0.003
	(0.003)	(0.002)	(0.002)
Subjective social status	0.105 ***	0.101 ***	0.101 ***
	(0.004)	(0.004)	(0.004)
Local GDP per capita	0.001	0.002 *	0.001
	(0.001)	(0.000)	(0.000)
Local income level	−0.060	−0.060	−0.064
	(0.052)	(0.054)	(0.053)
Security of the community (safe = 1)	0.090 ***	0.067 **	0.069 **
	(0.021)	(0.021)	(0.021)
Population size of the community	−0.001	0.001	−0.001
	(0.001)	(0.001)	(0.003)
Constant	3.422 ***	2.878 ***	3.253 ***
	(0.153)	(0.101)	(0.154)
Log likelihood	−18,567.688	−18,465.673	−18,460.670
ICC			
City level	0.020 ***	0.025 ***	0.020 ***
Community level	0.054 ***	0.053 ***	0.053 ***
R_1_^2^	0.159	0.167	0.171
*N*	15,501	15,501	15,501
Number of groups at the city level	123	123	123
Number of groups at the community level	391	391	391

Note. Standard errors in parentheses. ^+^, *, **, and *** significance at the 10, 5, 1 and 0.1% levels, respectively. The ICC coefficient describes how strongly units in the same group resemble each other. R_1_^2^ refers to the proportional reduction in residual variance at the individual level compared to the null mode.

**Table 4:** Robustness checks.

	(4) IV = Theil Index	(5) IV = P90/P50	(6) DV = Life Satisfaction	(7) Multilevel Logit Model	
				b	Odds Ratio
Gini coefficient			−0.549 **	−1.471 *	0.229
			(0.196)	(0.663)	[0.062, 0.841]
Theil index	−0.238 **				
	(0.086)				
P90/P50		−0.023 *			
		(0.0096)			
Neighbourhood social capital at the community level	−0.089 ***	−0.092 ***	−0.072 **	−0.232 *	0.793
	(0.024)	(0.024)	(0.022)	(0.093)	[0.661, 0.950]
Neighbourhood social capital at the individual level	0.048 ***	0.048 ***	0.044 ***	0.089 ***	1.093
	(0.003)	(0.003)	(0.003)	(0.016)	[1.057, 1.128]
Constant	3.027 ***	2.983 ***	2.677 ***	0.585 ***	
	(0.114)	(0.110)	(0.148)	(0.107)	
Individual controls	YES	YES	YES	YES	
Aggregate-level controls	YES	YES	YES	YES	
Log likelihood	−18,462.05	−18,462.755	−18,465.099	−3406.878	
ICC					
City level	0.021 ***	0.023 ***	0.018 ***	0.032 ***	
Community level	0.053 ***	0.053 ***	0.043 ***	0.108 ***	
R_1_^2^	0.170	0.169	0.178	-	
*N*	15,501	15,501	15,501	15,501	
Number of groups at the city level	123	123	123	123	
Number of groups at the community level	391	391	391	391	

Note. Standard errors in parentheses. ^+^, *, **, and *** significance at the 10, 5, 1 and 0.1% levels, respectively. The 95% confidence intervals in brackets. ICC describes how strongly units in the same group resemble each other. R_1_^2^ refers to the proportional reduction in residual variance at the individual level compared to the null mode. DV = dependent variable, IV = independent variable.

**Table 5:** Estimation results for the moderating effect of neighbourhood social capital.

	(8) IV = Gini Coefficient	(9) IV = Theil Index	(10) IV = P90/P50
Gini coefficient	−1.243 *		
	(0.586)		
Theil index		−0.576 *	
		(0.262)	
P90/P50			−0.090 *
			(0.038)
Neighbourhood social capital at the community level	0.009	−0.069	−0.133 *
	(0.144)	(0.0644)	(0.062)
Neighbourhood social capital at the individual level	0.005	0.029 ***	0.035 ***
	(0.017)	(0.008)	(0.006)
Gini coefficient × NSC_1	0.083 *		
	(0.035)		
Theil index × NSC_1		0.037 *	
		(0.015)	
P90/P50 × NSC_1			0.004 *
			(0.002)
Gini coefficient × NSC_2	−0.190		
	(0.285)		
Theil index × NSC_2		−0.038	
		(0.122)	
P90/P50 × NSC_2			0.013
			(0.018)
Constant	3.537 ***	3.189 ***	3.192 ***
	(0.306)	(0.157)	(0.164)
Individual controls	YES	YES	YES
Aggregate-level controls	YES	YES	YES
Log likelihood	−18,457.853	−18,459.003	−18,460.018
ICC			
City level	0.020 ***	0.022 ***	0.022 ***
Community level	0.053 ***	0.054 ***	0.053 ***
R_1_^2^	0.171	0.171	0.170
*N*	15,501	15,501	15,501
Number of groups at the city level	123	123	123
Number of groups at the community level	391	391	391

Note. Standard errors in parentheses. ^+^, *, **, and *** significance at the 10, 5, 1 and 0.1% levels, respectively. ICC describes how strongly units in the same group resemble each other. R_1_^2^ refers to the proportional reduction in residual variance at the individual level compared to the null mode. IV = independent variable, NSC_1 = neighbourhood social capital at the individual level, NSC_2 = neighbourhood social capital at the community level.

**Table 6:** The moderating effects for subsamples.

	Education		Income	
	(11) Low	(12) High	(13) Low	(14) High
Gini coefficient	−1.619 *	−0.438	−1.768 *	−0.735
	(0.679)	(0.879)	(0.707)	(0.705)
Neighbourhood social capital at the community level	−0.120	0.089	−0.071	0.078
	(0.164)	(0.204)	(0.166)	(0.171)
Neighbourhood social capital at the individual level	0.013	0.026	−0.003	0.017
	(0.023)	(0.032)	(0.025)	(0.025)
Gini coefficient × NSC_1	0.074 ^+^	0.030	0.095 ^+^	0.067
	(0.043)	(0.069)	(0.051)	(0.048)
Gini coefficient × NSC_2	0.045	−0.280	0.034	−0.357
	(0.322)	(0.413)	(0.329)	(0.336)
Constant	3.682 ***	3.164 ***	3.210 ***	3.326 ***
	(0.358)	(0.457)	(0.402)	(0.373)
Individual controls	YES	YES	YES	YES
Aggregate-level controls	YES	YES	YES	YES
Log likelihood	−13,012.741	−5454.330	−9135.433	−9342.952
ICC				
City level	0.018 ***	0.027 ***	0.023 ***	0.020 ***
Community level	0.058 ***	0.058 ***	0.053 ***	0.056 ***
R_1_^2^	0.156	0.211	0.202	0.148
*N*	10,838	4663	7773	7728
Number of groups at the city level	123	122	123	123
Number of groups at the community level	385	379	391	389

Note. Standard errors in parentheses. ^+^, *, **, and *** significance at the 10, 5, 1 and 0.1% levels, respectively. ICC describes how strongly units in the same group resemble each other. R_1_^2^ refers to the proportional reduction in residual variance at the individual level compared to the null mode. NSC_1 = neighbourhood social capital at the individual level, NSC_2 = neighbourhood social capital at the community level. Low-income group refers to respondents whose income level is lower than the average income of the city. High-income group refers to respondents whose income level is higher than or equal to the average income level of the city. Whether a respondent has a senior high school education or not is used as the criterion to distinguish high education level from low education level.

## Data Availability

Data used in this paper is from the China Labor-force Dynamics Survey (CLDS) by the Center for Social Science Survey at Sun Yat-sen University in Guangzhou, China. Please refer to http://css.sysu.edu.cn (accessed on 18 April 2020) for more information about the raw data. The authors appreciate the assistance in providing data by the institute; any errors are our own.
